# In vitro characterization of an osteoinductive biphasic calcium phosphate in combination with recombinant BMP2

**DOI:** 10.1186/s12903-016-0263-3

**Published:** 2016-08-02

**Authors:** Yang Shuang, Lin Yizhen, Yufeng Zhang, Masako Fujioka-Kobayashi, Anton Sculean, Richard J. Miron

**Affiliations:** 1 The State Key Laboratory Breeding Base of Basic Science of Stomatology (Hubei-MOST) & Key Laboratory of Oral Biomedicine Ministry of Education, School & Hospital of Stomatology, Wuhan University, 237 Luoyu Road, Wuhan, 430079 People’s Republic of China; 2 Department of Oral Implantology, School of Stomatology, Wuhan University, Wuhan, 430079 People’s Republic of China; 3 Department of Cranio-Maxillofacial Surgery, Bern University Hospital, Inselspital, Bern, 3010 Switzerland; 4 Department of Oral Surgery, Institute of Biomedical Sciences, Tokushima University, 3-18-15 Tokushima, Tokushima, Japan; 5 Department of Periodontology, School of Dental Medicine, University of Bern, Freiburgstrasse 7, Bern, 3010 Switzerland; 6 Department of Periodontology, College of Dental Medicine, Nova Southeastern University, Fort Lauderdale, FL 33328 USA; 7 Department of Oral Surgery and Stomatology, University of Bern, Bern, Switzerland

**Keywords:** Bone morphogenetic protein, Vivoss, BCP, Growth factors, Guided bone regeneration

## Abstract

**Background:**

The repair of alveolar bone defects with growth factors and bone grafting materials has played a pivotal role in modern dentistry. Recombinant human bone morphogenetic protein-2 (rhBMP2), an osteoinductive growth factor capable of cell recruitment and differentiation towards the osteoblast lineage, has been utilized in combination with various biomaterials to further enhance new bone formation. Recently, a group of novel biphasic calcium phosphate (BCP) bone grafting materials have been demonstrated to possess osteoinductive properties by demonstrating signs of ectopic bone formation. The aim of the present study was to study the effects of rhBMP2 in combination with osteoinductive BCP bone grafts on osteoblast cell behaviour.

**Methods:**

MC3T3-E1 pre-osteoblasts were seeded on 1) control tissue culture plastic, 2) 10 mg of BCP alone, 3) 100 ng rhBMP2, and 4) 100 ng rhBMP2+ 10 mg of BCP and analyzed for cell recruitment via a Transwell chamber, proliferation via an MTS assay and differentiation as assessed by alkaline phosphatase (ALP) activity, alizarin red staining and real-time PCR for osteoblast differentiation markers including Runx2, collagen1, ALP, and osteocalcin (OCN).

**Results:**

rhBMP2 was able to significantly upregulate cell recruitment whereas the addition of BCP as well as BCP alone had no additional ability to improve osteoblast recruitment. Both BCP and rhBMP2 were able to significantly increase cell proliferation at 3 and 5 days post seeding and cell number was further enhanced when rhBMP2 was combined with BCP. In addition, the combination of rhBMP2 with BCP significantly improved ALP activity at 7 and 14 days post seeding, alizarin red staining at 14 days, and mRNA levels of Runx2, ALP and osteocalcin when compared to cells seeded with rhBMP2 alone or BCP alone.

**Conclusions:**

The results from the present study demonstrate that 1) the osteoinductive potential of BCP bone particles is equally as osteopromotive as rhBMP2 on in vitro osteoblast differentiation and 2) BCP particles in combination with rhBMP2 is able to further increase the osteopromotive differentiation of osteoblasts in vitro when compared to either rhBMP2 alone or BCP alone. Future animal testing is further required to investigate this combination approach on new bone formation.

## Background

The repair of alveolar bone defects with growth factors and bone grafting materials have played a pivotal role in modern dentistry [1–4]. Although autogenous bone has been considered the gold standard of bone grafting materials for a number of years due to its excellent combination of osteogenesis (contains living progenitor), osteoinduction (contains a number of growth factors capable of recruiting progenitor cells and/or form ectopic bone formation) and osteoconduction (able to support three-dimensional tissue ingrowth and future new bone formation) [5–8]. Despite these advantages, drawbacks including additional donor site morbidity, limited harvesting supply within the oral cavity and additional surgical time and costs have necessitated alternatives. These include allografts harvested from human donors, xenografts harvested from an animal donor and a wide variety of synthetically fabricated bone grafts made from hydroxyapatite, tri-calcium phosphate, biphasic calcium phosphate and bioactive glasses [9–13].

Currently, one area of research that has been highly investigated in recent years is the field of osteoinductive biomaterials [7]. Originally, osteoinduction was described as the ability for an undifferentiated progenitor cell to auto-induce down the osteoblast lineage [14]. Much of the original findings were developed by Urist and Strates who extracted a complex of bone morphogenetic proteins (BMPs) from demineralized bone matrix [15]. Since the mid 1970s, the only FDA approved commercially available biomaterials with ‘osteoinductive potential’ are limited to demineralized freeze-dried bone allograft (DFDBA) and BMP2 [7]. More recently however, it has been shown that synthetically fabricated bone grafts made from biphasic calcium phosphate (BCP) materials sintered at low temperatures have shown the potential for osteoinduction by demonstrating ectopic bone formation when materials were implanted in extra-skeletal sites (either muscle or epithelial tissues) [16, 17]. As the ability for these materials to guide mesenchymal progenitor cells down the osteoblast lineage without the use of inductive growth factors such as BMP2, our laboratory has become increasingly interested in their potential as future regenerative materials. One of the main thoughts contributing to their osteoinductive potential has been proposed to be highly regulated from cells derived from the monocyte lineage including either macrophages or osteoclasts [18]. Currently, an array of recent research has been carried out investigating these materials and their ability to induce ectopic bone formation [19–23].

A recently published article from our group investigated the in vitro characterization of these novel osteoinductive scaffolds. It was shown that an equal ability to promote rapid transformation of mesenchymal progenitor cells towards the osteoblast lineage was found between BCP bone grafts and autogenous bone by improving mRNA levels of runx2, collagen 1, alkaline phosphatase and osteocalcin [24]. These novel BCP bone grafts differ from previous versions of BCP as they contain more micro- and nano-topographies which are spontaneously able to induce ectopic bone formation and are thought to contribute more rapidly to new bone formation. Thus, it becomes a hypothesis that the combination of BCP and rhBMP2 may potentially be combined to improve new bone formation. The mode of action of BMP2 is to recruit progenitor cells to defect sites and guide their differentiation towards the osteoblast lineage [25]. In contrast, although these novel BCP scaffolds have previously reported no ability to recruit cells, they auto-induce a form of osteoinduction by completely different mechanisms guiding mesenchymal stem cell (MSC) differentiation towards the osteoblast lineage likely due material surface cues guided by topography and possibly dissolution of the material. Due to the different mechanisms by which BMP2 and BCP scaffolds are able to induce osteoinduction, it thus becomes of interest to combine both materials to determine if the bone inducing capabilities of BMP2 can be enhanced via its combination with osteoinductive BCP scaffolds. Therefore, the aim of the present study was to combine BMP2 with BCP bone grafts and test their ability on in vitro cell behaviour when compared to BMP2 alone, BCP alone as well as to tissue culture plastic alone. Cells were compared for cell recruitment, cell proliferation and cell differentiation as assessed by alkaline phosphatase activity, alizarin red staining for mineralization as well as real-time PCR for osteoblast differentiation markers including Runx2, collagen 1, alkaline phosphatase and osteocalcin.

## Methods

### BMP2 gene sequence and biphasic calcium phosphate bone graft

**Fig. 1:**
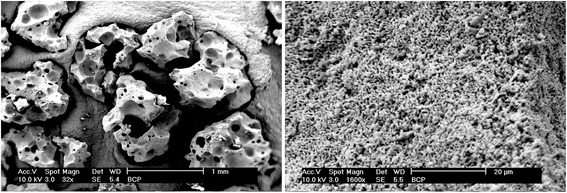
Scanning electron microscopy image of BCP scaffolds at low and high magnification demonstrated many macro- and nano-topographies

BMP2 was kindly provided by Jiuyuan biotech company (Hangzhou, China). The amino acid sequence was fabricated as follows: Met Lys Lys Leu Lys Ser Ser Cys Lys Arg His Pro Leu Tyr Val Asp Phe Ser Asp Val Gly Trp Asn Asp Trp Ile Val Ala Pro Pro Gly Tyr His Ala Phe Tyr Cys His Gly Glu Cys Pro Phe Pro Leu Ala Asp His Leu Asn Ser Thr Asn His Ala Ile Val Gln Thr Leu Val Asn Ser Val Asn Ser Lys Ile Pro Lys Ala Cys Cys Val Pro Thr Glu Leu Ser Ala Ile Ser Met Leu Tyr Leu Asp Glu Asn Glu Lys Val Val Leu Lys Asn Tyr Gln Asp Met Val Val Glu Gly Cys Gly Cys Arg for a protein chain of 108 amino acids fabricated from E. coli. The concentration of rhBMP2 used in this study (100 ng/ml) was selected based on previous concentration dependent experiments utilizing rhBMP2 (unpublished data). The BCP grafting particles were kindly provided by Straumann AG (Vivoss, Basel, Switzerland) and are composed of hydroxyapatite and beta-tricalcium phosphate in a 10:90 ratio with a micro-porous surface structure and a crystallinity ≥80 %. A scanning electron microscopy (SEM) image of the bone graft can be found in Fig. 1 demonstrating its macro and micro-rough surface. Previous studies have described these bone grafts as inducers of ectopic bone formation [18–23, 26, 27].

### Two-dimensional migration assay

MC3T3-E1 pre-osteoblast cell line (ThermoScientific, Waltham, Massachusetts, United States) was used for this study. All cells were cultured in tissue culture flasks using α-MEM supplemented with antibiotics (100 μg/ml penicillin G, Sigma-Aldrich; 50 μg/ml gentamicin, Sigma-Aldrich; 3 mg/ml amphotericin B, Gibco, Grand Island, NY, USA and 10 % fetal bovine serum). No additional osteoblast differentiation media was added for experiments to fully investigate the potential for each group to influence MC3T3-E1 pre-osteoblast differentiation towards mineralize-producing osteoblasts. Cells were removed from the tissue culture plastic using a trypsin solution [0.25 % trypsin (Gibco), 0.1 % glucose, citrate-saline buffer (pH 7.8)]. The migration assay of cells was performed with a Transwell chamber using a 24-well plate and polycarbonate filters (Transwell Costar, Corning, Acton, MA) with a pore size of 8 μm as previously described [28]. Ten thousand MC3T3-E1 cells in 50 μl DMEM were seeded in the upper compartment. Samples included 1) control tissue culture plastic, 2) 10 mg of BCP alone, 3) rhBMP2 at concentrations of 100 ng/ml and 4) rhBMP2 at a concentration of 100 ng/ml + 10 mg BCP were seeded into the lower compartment. The cells were allowed to migrate for 24 h at 37 °C in a humidified 5 % CO_2_ atmosphere. The filter was then removed, cells were fixed in 4 % formaldehyde for 20 min and washed in PBS, permeabilized in 0.5 % Triton-X for 15 min, and washed again in phosphate buffered solution (PBS). After washings, filters were incubated for 1 h at room temperature with Alexa Fluor^TM^ 568 phalloidin (1:50 dilution; Invitrogen A12380) diluted in 0.1 % PBS/BSA. Samples were examined by confocal microscopy. Non-migrated cells on the upper side were eliminated by rinsing the filter with cold PBS and scraping with a rubber wiper. The remaining migrated cells on the lower side of the filter were counted in nine random fields per filter (×100 magnification). All samples were performed in triplicate with three independent experiments performed and normalized to control samples.

### Cell growth assay

To investigate the effect on cell number of MC3T3-E1, CCK-8 assay for 1) control tissue culture plastic, 2) 10 mg of BCP alone, 3) rhBMP2 at concentrations of 100 ng/ml and 4) rhBMP2 at a concentration of 100 ng/ml + 10 mg BCP was performed as previously described [29]. Briefly, cells were seeded in 96-well plates at a density of 5000 cells/well. At time points 1, 3 and 5 days, the CCK-8 assay was performed by adding 10 μL of the CCK-8 solution (Dojindo Molecular Technologies, Inc. Japan) to each well and incubating for 1.5 h according to manufacturer’s protocol. The absorbance was measured at λ = 450 nm on a plate reader. Results were demonstrated as the absorbance of each experimental well minus the optical density value of blank wells. All samples were repeated in triplicate with three independent experiments.

### Alkaline phosphatase activity

Alkaline Phosphatase activity was analyzed colorimetrically using alkaline phosphatase assay kit (Nanjing Jiancheng Bioengineering Insitute, China) using a starting seeding density of 50,000 cells per 24 well dish as previously described [30, 31]. At time point of 7 and 14 days, cells were washed three times with PBS and solubilized in 0.1 % Triton X-100 at 4 °C for 1 h. After sonication and centrifugation, ALP activity in the supernatant was determined colorimetrically using readings OD405/OD562. Samples were run in triplicate with three independent experiments performed and normalized to total DNA content.

### Real-time PCR

MC3T3-E1 cells were seeded at a starting seeding density of 50,000 cells per 24 well dish and cultured for 7 and 14 days as previously described [31, 32]. The effects of 1) control tissue culture plastic, 2) 10 mg of BCP alone, 3) rhBMP2 at concentrations of 100 ng/ml and 4) rhBMP2 at a concentration of 100 ng/ml + 10 mg BCP were investigated on four osteogenic-related gene expression including runt-related transcription factor 2 (Runx 2), collagen I (COLI), alkaline phosphatase (ALP) and osteocalcin (OCN). Total RNA was extracted from MC3T3-E1 cells by Trizol reagent (TriPure Isolation Reagent, Roche Applied Science, Germany) according to the manufacturer’s instructions. The concentration and quality of the total RNA samples was carried out by Nanodrop (Thermo Fisher Scientific Inc.). Complementary DNA was synthesized from 2 μg of total RNA using RevertAidTm First Strand cDNA Synthesis Kit (Fermentas) following the manufacturer’s protocol. RT-qPCR was performed in 20 μL reactions containing 10 μl SYBR Green Master Mix (Roche Applied Science, Germany), 0.6 μL (10 μM) of each forward and reverse primer for each gene of interest, 2 μL of cDNA template and 6.8 μL water. Glyceraldehyde-3-phosphate-dehydrogenase (GAPDH), a reference gene, was used as a control. The reaction was carried out using an ABI Prism 7000 Sequence Detection System (Applied Biosystems), and the PCR amplification run for 40 cycles. To validate specific amplicon amplification without genomic DNA contamination, melting curve analysis was performed and the single apex appeared around the annealing temperature. Relative expression levels for each desired gene were normalized against the Ct value of GAPDH and determined by using the delta Ct method. All samples were determined in triplicate for three independent studies.

### Alizarin red quantification

Alizarin red staining was performed to determine the presence of extracellular matrix mineralization after 14 days. MC3T3-E1 cells were seeded at a density of 50’000 cells per 24 well culture dish containing 1) control tissue culture plastic, 2) 10 mg of BCP alone, 3) rhBMP2 at concentrations of 100 ng/ml and 4) rhBMP2 at a concentration of 100 ng/ml + 10 mg BCP. After 14 days, cells were fixed in 96 % ethanol for 15 min and stained with 0.2 % alizarin red solution in water (pH 6.4) at room temperature for 1 h and visualized under light microscopy. Thereafter, alizarin red quantification was dissolved using 200 μl of 1 % cetylpyridinium chloride (dissolved with double distilled water) at room temperature for 4 h. Then 100 μl solution was transferred to 96-well plate to text OD 560. Thereafter samples were normalized to control samples which included first BCP bone grafts alone without cells (for BCP group ad BCP + rhBMP2 group only), and thereafter to control tissue culture plastic samples as previously described [5].

### Statistical analysis

All data analysis was performed using SPSS 17.0 software and statistically significant values were adopted as *p* < 0.05. Mean and standard deviation (SD) were analyzed using one-way ANOVA with a post hoc *t*-test. Use of ANOVA assumes that the data are normally distributed, and that the variances of the different groups do not differ significantly.

## Results

### Migration assay

**Fig. 2:**
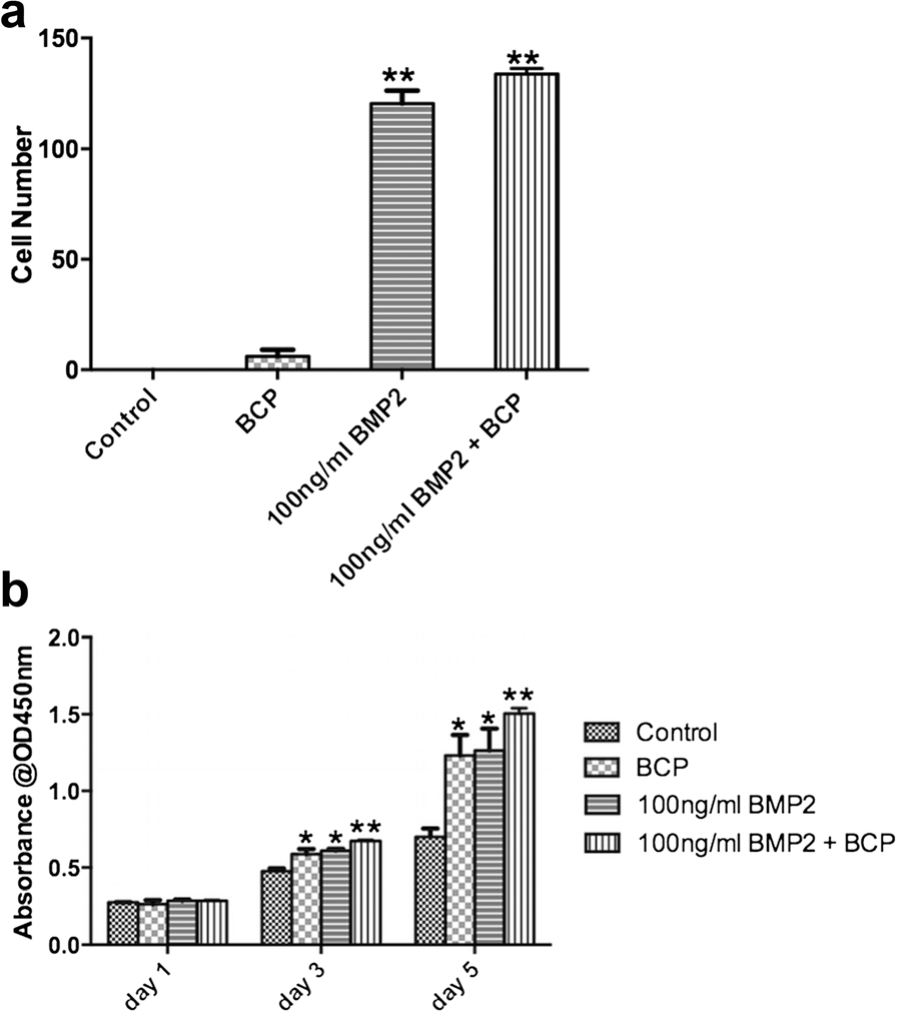
**a** Cell recruitment assay demonstrated that 100 ng/ml of rhBMP2 is significantly able to improve MC3T3-E1 cell recruitment (Cell number per field of view). **b** Proliferation assay of MC3T3-E1 cells seeded at 1, 3 and 5 days post seeding demonstrated that no further advantage of combining rhBMP2 with BCP or BCP alone could be observed cell number of MC3T3-E1 cells demonstrated that the combination of rhBMP2 + BCP significantly increased numbers at 3 and 5 days post seeding when compared to rhBMP2 alone or BCP alone (*, *p* < 0.05, denotes significant difference between group and control, ** *p* <0.05, denotes significantly higher than all other groups)

A transwell assay was used to investigate the effects of rhBMP2 and BCP grafts on cell recruitment (Fig. 2a). It was first found that BCP alone had no ability to recruit MC3T3-E1 cells when compared to control samples whereas 100 ng/ml of rhBMP2 was able to significantly upregulate cell recruitment (*p* < 0.05). The combination approach utilizing BCP with 100 ng/ml of rhBMP2 was unable to further increase cell recruitment when compared to rhBMP2 alone (Fig. 2a).

### Cell growth assay

The proliferation of MC3T3-E1 cells was then investigated to determine the effects of each group on cell number (Fig. 2b). It was found that at 1 day post seeding, all cells adhered and were present in similar numbers. By 3 days however, cells that were seeded in the presence of 100 ng/ml of rhBMP2 or BCP demonstrated significantly higher cell numbers when compared to control samples (*p* < 0.05, Fig. 2b). Furthermore, the combination of rhBMP2 with BCP displayed significantly higher levels then all other groups (**p < 0.01). At 5 days post seeding, a significant increase between BCP and control samples could be observed. Additionally, rhBMP2 further stimulated cell numbers when compared to control, and the combination of BCP + rhBMP2 led to significantly higher cell number when compared to all other treatment groups (*p* < 0.05, Fig. 2b).

### Differentiation assays

**Fig. 3:**
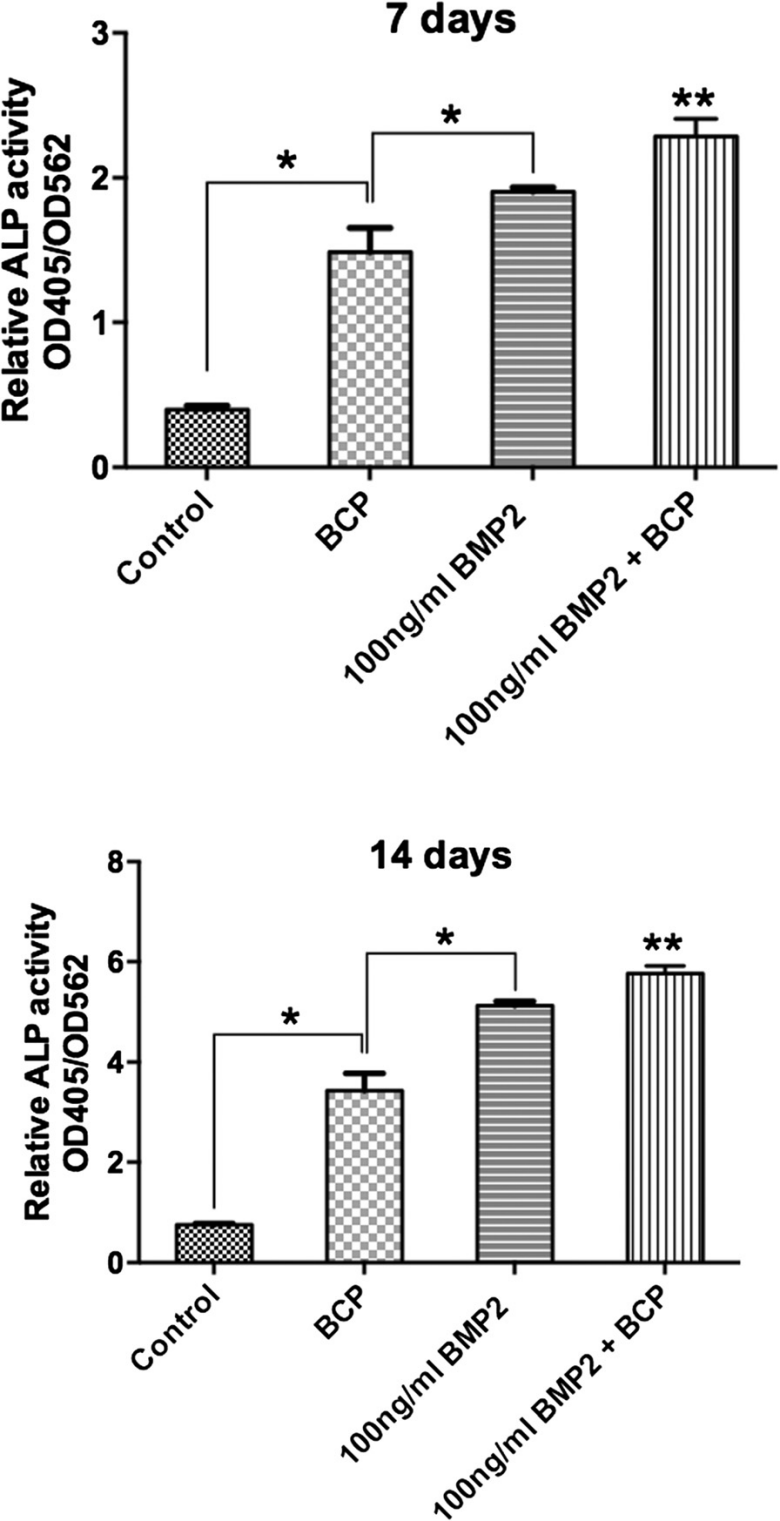
ALP activity was significantly increased at 7 and 14 days post seeding for samples seeded with 1) control tissue culture plastic, 2) BCP alone 3) 100 ng rhBMP2, and 4) 100 ng rhBMP2 + BCP (*p < 0.05, denotes significant differences between groups, ** *p* <0.05, denotes significantly higher than all other groups)

**Fig. 4:**
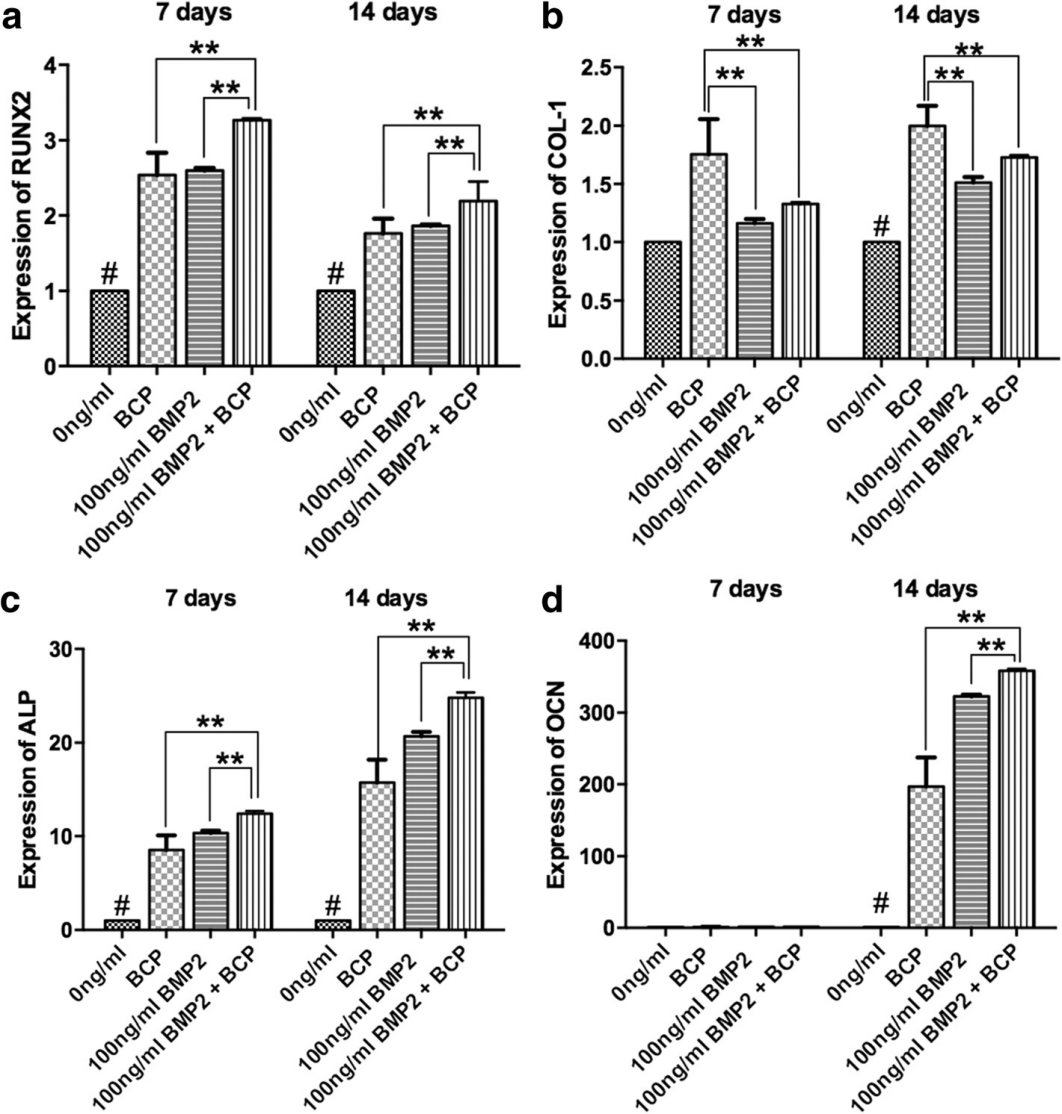
mRNA levels of **a** RUNX2, **b** COL-1, **c** ALP and **d** OCN for MC3T3-E1 cells seeded on 1) control tissue culture plastic, 2) BCP alone 3) 100 ng rhBMP2, and 4) 100 ng rhBMP2 + BCP at 7 and 14 days post seeding (*p < 0.05, denotes significance between groups, # *p* < 0.05, denotes significantly lower than all other treatment groups, ** *p* < 0.05, denotes significantly higher than all other treatment groups)

**Fig. 5:**
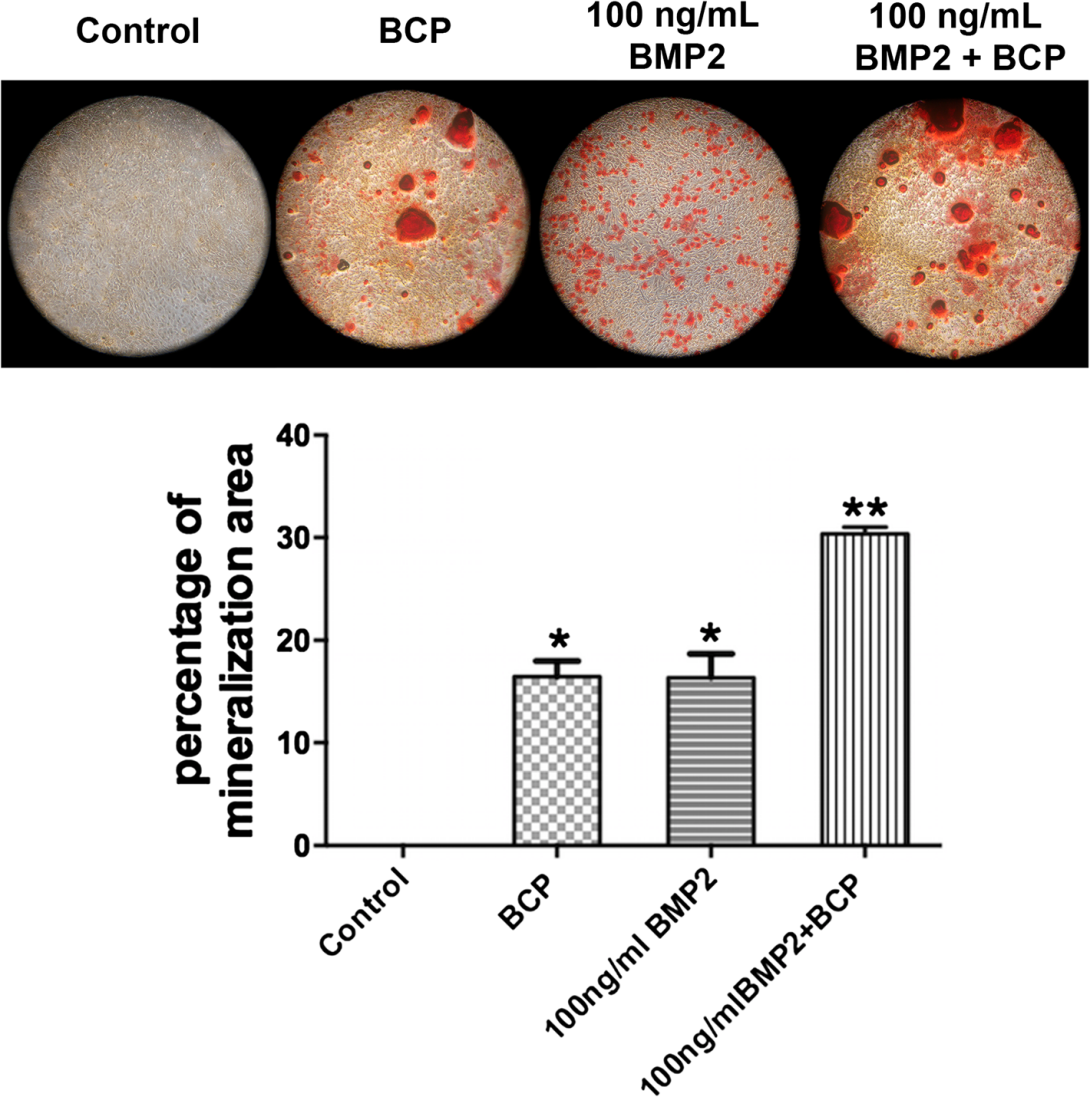
**a** Qualitative analysis of alizarin red staining to demonstrate areas of mineralization in MC3T3-E1 cells seeded on 1) control tissue culture plastic, 2) BCP alone, 3) 100 ng rhBMP2, and 4) 100 ng rhBMP2 + BCP at 14 days post seeding. **b** Percentage of mineralization area from alizarin red staining (* *p* < 0.05 denotes significant difference between group and control, ** *p* <0.05, denotes significantly higher than all other treatment)

In order to assess osteoblast differentiation, alkaline phosphatase activity, alizarin red staining and mRNA levels of osteoblast differentiation markers were investigated (Figs. 3, 4 and 5). It was first observed that the use of both BCP and rhBMP2 significantly increased ALP activity over three fold when compared to control samples at both 7 and 14 days post seeding (Fig. 3). Moreover, rhBMP2 demonstrated significantly higher ALP expression when compared to BCP at both time points (Fig. 3). The combination of BCP scaffolds with rhBMP2 led to significantly higher ALP activity when compared to all other treatment groups at both 7 and 14 days (Fig. 3).

Real time PCR was then investigated to analyze the following four genes Runx2, COL-1, ALP and OCN (Fig. 4). It was found that the addition of rhBMP2 or BCP was able to significantly increase Runx2 expression two fold at 7 and 14 days (Fig. 4a). Furthermore, group containing rhBMP2 + BCP led to significantly higher levels when compared to all other groups at 7 days (Fig. 4a). Expression of COL-1 showed that cell seeded with BCP demonstrated significantly higher levels when compared to all other treatment modalities at both 7 and 14 days (Fig. 4b). ALP activity was significantly increased approximately ten fold for BCP and rhBMP2 at both time points with rhBMP2 + BCP demonstrating significantly higher levels when compared to all other groups at both 7 and 14 days (Fig. 4c). No significant changes for OCN expression was observed at 7 days (Fig. 4d), however, at 14 days, over a 150 fold increase could be observed in BCP and rhBMP2 groups when compared to controls (Fig. 4d). Furthermore, rhBMP2 + BCP group demonstrated significantly higher OCN expression when compared to all other modalities (Fig. 4d).

Alizarin red staining was then utilized to visualize the mineralization potential of each sample (Fig. 5). In the absence of osteoblast differentiation media, it was observed that control samples had no ability to produce mineralization following 14 days culture (Fig. 5). In the group receiving rhBMP2, evidence of mineralization was apparent via alizarin red staining (Fig. 5, red dots). The samples with BCP alone also demonstrated signs of mineralization potential primarily found in cells around the bone grafting particles. The samples containing both BCP and rhBMP2 demonstrated signs of elevated staining with apparent staining around the bone grafting particles (Fig. 5). Quantification of alizarin red data demonstrated that the combination of rhBMP2 with BCP significantly increased mineralization when compared to all other groups (Fig. 5).

## Discussion

The aim of the present study was to investigate whether the combination of rhBMP2 could further enhance osteoblast activity by combining its osteoinductive properties with novel BCP bone grafts. These new grafts have been shown to be osteoinductive by demonstrating signs of ectopic bone formation in various animal models [16, 17]. Recent investigations on their mode of action have suggested that these grafts promote rapid transformation from mesenchymal progenitor cell to osteoblasts, a phenomenon that appears to be guided by surface topography and dissolution of the material composition. However, a complete understanding is still lacking [18, 33–38]. While a wide variety of work is still necessary to better understand the main factors driving their ability to enhance ectopic bone formation, recent animal models confirm that these synthetically fabricated grafts are equally or more potent at forming new bone when compared to other synthetic bone grafting materials commercially available [39, 40]. Thus, it becomes of interest to design new strategies to implement these bone grafts with osteoinductive growth factors to further enhance new bone formation.

One of the key principles in designing new biomaterials involves key understandings of the biomaterial characteristics necessary to further enhance new bone formation. As such, guidelines have previously been established in a recent review article published by our group [7]. In order to facilitate osteoinduction, three key principals are necessary for improved osteoinductive potential of biomaterials [7]. Firstly, an ability to recruit mesenchymal progenitor cells to defect sites and biomaterial surfaces is crucial to the future success of their regenerative potential [7]. Secondly, the ability for the biomaterial to guide rapid differentiation of cells down the osteoblast lineage is necessary [7]. Lastly, all osteoinductive biomaterials should demonstrate the potential to form ectopic bone formation per guidelines established by Urist et al. in the mid 1960s [14, 15]. Thus, the combination of rhBMP2 with BCP scaffolds provides evidence of these concepts by providing additional biological rational. While rhBMP2 is able to improve principle 1 by recruiting progenitor cells to defect sites, once the cells have reached the material surface BCP scaffolds are then able to facilitate their differentiation towards the osteoblast lineage (principle 2). Furthermore, both of these materials alone are able to induce ectopic bone formation in extra-skeletal sites (principal 3).

The results from the present study demonstrate that rhBMP2 is able to recruit progenitor cells as found in our transwell assay (Fig. 2). The addition of BCP did not improve cell recruitment nor did BCP alone. This is likely due to the fact that BCP scaffolds contain no growth factors or chemokines able to recruit progenitor cells. When cell proliferation was analyzed, it was found that the combination of bone rhBMP + BCP significantly enhanced cell numbers at both 3 and 5 days post seeding when compared to rhBMP2 alone or to BCP alone (Fig. 2b). Thus, the use of bioactive biomaterials further supported the proliferation of cells and that their combination did provide additional benefit. However and more notably, most of the drastic differences were observed when cell differentiation was analyzed. It was found in the present study that ALP activity was induced over three fold with either BCP alone or rhBMP2 alone at 7 and 14 days post seeding and this could be further enhanced when their combination was utilized (Fig. 3). Not surprisingly, all genes associated with osteoblast differentiation were further significantly increased when mRNA levels were assessed by real-time PCR for groups including either rhBMP2, BCP or a combination of rhBMP2 + BCP. It must also be pointed out that under the present in vitro setting, the data indicate that the use of rhBMP2 alone seemed to favour osteoblast differentiation more so then when BCP alone was utilized. The results clearly point to the osteoinductive ability of rhBMP2 on osteoblast activity. From a clinical perspective, it must be noted that the use of a bone grafting material provides significant other advantages apart from cell activity which include maintaining provisional space for defect healing as well as improving blood clot formation; two important factors in defect wound healing [41].

Interestingly, while genes encoding Runx2 and collagen1 at 7 and 14 days post seeding demonstrated at most a 2–3 fold upregulation across all tested experimental groups, ALP activity demonstrated a marked ten-fold increase at 7 days post seeding, and a 20 fold increase at 14 days post-seeding (Fig. 4). Furthermore, while OCN was elevated approximately 1.5 fold at 7 days post seeding, the gene expression of OCN raised over 200 fold by 14 days post-seeding for groups containing rhBMP2 (Fig. 4d). OCN is largely considered a late differentiation marker of osteoblasts. This finding is likely indicated that MC3T3-E1 pre-osteoblasts had not undergone full differentiation by 7 days, and that by 14 days had reached full maturity. Thus when the fully matured cells where compared to control undifferentiated cells which were cultured on cell culture plastic without rhBMP2 or BCP, it becomes apparent that these cells are not expressing OCN in high levels.

Interesting results were observed for alizarin red staining (Fig. 5). No staining was observed in control samples further confirming that these cells had not undergone cell differentiation and thus had no apparent ability to produce mineralized tissue in vitro. Cells that were cultured with rhBMP2 on the other hand produced droplets of alizarin red staining which were not found in levels consistent throughout the entire culture surface but instead were found in cluster regions which expressed highly mineralized areas (Fig. 5, rhBMP2 alone sample). It was further interesting to observe that in samples cultured with BCP bone grafting materials, much of the staining was observed around the bone grafting particles (Fig. 5) Thus, it becomes evident that either the dissolution of the bone grafting material is influencing the surrounding cell’s ability to differentiate and produce mineralized tissue, or that somehow cells adjacent to the material surface are able to communicate to adjacent cells via cell-cell communication molecules such as connexins and gap junctions able to influence osteoblast differentiation. A further understanding of these observed results could provide additional cues as to how these scaffolds are able to further increase osteoblast differentiation.

One area of research that remains to be addressed is the mechanism by which these novel osteoinductive bone grafts are able to stimulate ectopic bone formation. Recent studies have revealed that the immune system and the field of osteoimmunology is likely to provide the key features to certain synthetic bone grafts being osteoinductive [18, 20, 27]. An animal macrophage knock-out system revealed that the elimination of macrophage completely abolished the osteoinductive potential of these BCP grafts [18, 20, 27]. This special subset of macrophages which has been refered to in the literature as ‘OsteoMacs’ (short for osteal macrophages) is currently a key area of research focusing on their possible role in guiding new bone regeneration around certain classes of bone biomaterials [42]. While data exist supporting their involvement to date, further investigation into new strategies to fully reveal their implication in bone formation will likely provide key valuable insights into future development of osteoinductive bone biomaterials since monocytes/macrophages are one of the first cell-type in contact with biomaterials and likely govern their future integration into host tissues.

## Conclusions

The results from the present study demonstrate that rhBMP2 was able to increase cell recruitment whereas its combination with BCP did not provide any additional benefit. Cell numbers were significantly higher at 3 and 5 days post seeding for the combination of rhBMP2 + BCP when compared to rhBMP2 alone and BCP alone. Interestingly, it was observed that these new BCP bone grafts were equally as osteopromotive when compared to rhBMP2 on in vitro osteoblast differentiation. Furthermore, the combination of both biomaterials had a further significant increase on cell differentiation as assessed by real-time PCR, ALP activity and alizarin red staining. The results from the present study suggest that the combination of both materials may improve the speed and quality of new bone formation in vivo, however future animal testing is required to validate this hypothesis.

## Abbreviations

ALP, alkaline phosphatase; BCP, biphasic calcium phosphate; BSP, bone sialoprotein; GAPDH, glyceraldehyde 3-phosphate dehydrogenase; MSC, mesenchymal stem cell; OCN, osteocalcin; PBS, phosphate buffered solution; rhBMP2, recombinant human bone morphogenetic protein 2; Runx2, runt-related transcription factor 2; SEM, scanning electron microscopy

## References

[1] Nokhbehsaim M, Deschner B, Winter J, Bourauel C, Rath B, Jager A, Jepsen S, Deschner J (2011). Interactions of regenerative, inflammatory and biomechanical signals on bone morphogenetic protein-2 in periodontal ligament cells. J Periodontal Res.

[2] Stavropoulos A, Wikesjo UM (2012). Growth and differentiation factors for periodontal regeneration: a review on factors with clinical testing. J Periodontal Res.

[3] Stancoven BW, Lee J, Dixon DR, McPherson JC, Bisch FC, Wikesjo UM, Susin C (2013). Effect of bone morphogenetic protein-2, demineralized bone matrix and systemic parathyroid hormone (1–34) on local bone formation in a rat calvaria critical-size defect model. J Periodontal Res.

[4] Yen CC, Tu YK, Chen TH, Lu HK (2014). Comparison of treatment effects of guided tissue regeneration on infrabony lesions between animal and human studies: a systematic review and meta-analysis. J Periodontal Res.

[5] Miron RJ, Hedbom E, Saulacic N, Zhang Y, Sculean A, Bosshardt DD, Buser D (2011). Osteogenic potential of autogenous bone grafts harvested with four different surgical techniques. J Dent Res.

[6] Miron RJ, Gruber R, Hedbom E, Saulacic N, Zhang Y, Sculean A, Bosshardt DD, Buser D (2013). Impact of bone harvesting techniques on cell viability and the release of growth factors of autografts. Clin Implant Dent Relat Res.

[7] Miron RJ, Zhang YF (2012). Osteoinduction: a review of old concepts with new standards. J Dent Res.

[8] Albrektsson T, Johansson C (2001). Osteoinduction, osteoconduction and osseointegration. Eur Spine J.

[9] Lekovic V, Milinkovic I, Aleksic Z, Jankovic S, Stankovic P, Kenney EB, Camargo PM (2012). Platelet-rich fibrin and bovine porous bone mineral vs. platelet-rich fibrin in the treatment of intrabony periodontal defects. J Periodontal Res.

[10] Kim MS, Lee JS, Shin HK, Kim JS, Yun JH, Cho KS. Prospective randomized, controlled trial of sinus grafting using Escherichia-coli-produced rhBMP-2 with a biphasic calcium phosphate carrier compared to deproteinized bovine bone. Clin Oral Implants Res. 2015;26(12):1361-8. doi:10.1111/clr.12471. 10.1111/clr.12471 25186180

[11] Lee EU, Lim HC, Hong JY, Lee JS, Jung UW, Choi SH. Bone regenerative efficacy of biphasic calcium phosphate collagen composite as a carrier of rhBMP-2. Clin Oral Implants Res. 2015. doi:10.1111/clr.12568. [Epub ahead of print]. 10.1111/clr.12568 25675839

[12] Matsuura T, Akizuki T, Hoshi S, Ikawa T, Kinoshita A, Sunaga M, Oda S, Kuboki Y, Izumi Y. Effect of a tunnel-structured beta-tricalcium phosphate graft material on periodontal regeneration: a pilot study in a canine one-wall intrabony defect model. J Periodontal Res. 2015;50(3):347-55. 10.1111/jre.12213 25040655

[13] Yoshida T, Miyaji H, Otani K, Inoue K, Nakane K, Nishimura H, Ibara A, Shimada A, Ogawa K, Nishida E (2015). Bone augmentation using a highly porous PLGA/beta-TCP scaffold containing fibroblast growth factor-2. J Periodontal Res.

[14] Urist MR (1965). Bone: formation by autoinduction. Science (New York, NY.

[15] Urist MR, Strates BS (1971). Bone morphogenetic protein. J Dent Res.

[16] Yuan H, Kurashina K, de Bruijn JD, Li Y, De Groot K, Zhang X (1999). A preliminary study on osteoinduction of two kinds of calcium phosphate ceramics. Biomaterials.

[17] Yuan H, Fernandes H, Habibovic P, de Boer J, Barradas AMC, de Ruiter A, Walsh WR, van Blitterswijk CA, de Bruijn JD (2010). Osteoinductive ceramics as a synthetic alternative to autologous bone grafting. Proc Natl Acad Sci.

[18] Davison NL, Gamblin AL, Layrolle P, Yuan H, de Bruijn JD, Barrere-de Groot F (2014). Liposomal clodronate inhibition of osteoclastogenesis and osteoinduction by submicrostructured beta-tricalcium phosphate. Biomaterials.

[19] Barbieri D, de Bruijn JD, van Blitterswijk CA, Yuan H (2014). On the horizon: instructive nanomaterials hold the potential to mimic tissue complexity. IEEE Pulse.

[20] Davison NL, ten Harkel B, Schoenmaker T, Luo X, Yuan H, Everts V, Barrere-de Groot F, de Bruijn JD (2014). Osteoclast resorption of beta-tricalcium phosphate controlled by surface architecture. Biomaterials.

[21] Wang L, Barbieri D, Zhou H, de Bruijn JD, Bao C, Yuan H (2015). Effect of particle size on osteoinductive potential of microstructured biphasic calcium phosphate ceramic. J Biomed Mater Res A.

[22] Zhang J, Barbieri D, ten Hoopen H, de Bruijn JD, van Blitterswijk CA, Yuan H (2015). Microporous calcium phosphate ceramics driving osteogenesis through surface architecture. J Biomed Mater Res A.

[23] Zhang J, Luo X, Barbieri D, Barradas AM, de Bruijn JD, van Blitterswijk CA, Yuan H (2014). The size of surface microstructures as an osteogenic factor in calcium phosphate ceramics. Acta Biomater.

[24] Miron RJ, Sculean A, Shuang Y, Bosshardt DD, Gruber R, Buser D, Chandad F, Zhang Y. Osteoinductive potential of a novel biphasic calcium phosphate bone graft in comparison with autographs, xenografts, and DFDBA. Clin Oral Implants Res. 2016;27(6):668-75. 10.1111/clr.12647 26227281

[25] Ryoo H-M, Lee M-H, Kim Y-J (2006). Critical molecular switches involved in BMP-2-induced osteogenic differentiation of mesenchymal cells. Gene.

[26] Davison NL, Luo X, Schoenmaker T, Everts V, Yuan H, Barrere-de Groot F, de Bruijn JD (2014). Submicron-scale surface architecture of tricalcium phosphate directs osteogenesis in vitro and in vivo. Eur Cell Mater.

[27] Davison NL, Su J, Yuan H, van den Beucken JJ, de Bruijn JD, Barrere-de Groot F (2015). Influence of surface microstructure and chemistry on osteoinduction and osteoclastogenesis by biphasic calcium phosphate discs. Eur Cell Mater.

[28] Zhang Y, Ma Y, Wu C, Miron RJ, Cheng X. Platelet-derived growth factor BB gene-released scaffolds: biosynthesis and characterization. J Tissue Eng Regen Med. 2013. doi: doi:10.1002/term.1825. [Epub ahead of print]. 10.1002/term.1825 24130059

[29] Zhang Y, Wei L, Chang J, Miron RJ, Shi B, Yi S, Wu C (2013). Strontium-incorporated mesoporous bioactive glass scaffolds stimulating in vitro proliferation and differentiation of bone marrow stromal cells and in vivo regeneration of osteoporotic bone defects. J Mater Chem B.

[30] Miron RJ, Hedbom E, Ruggiero S, Bosshardt DD, Zhang Y, Mauth C, Gemperli AC, Iizuka T, Buser D, Sculean A (2011). Premature osteoblast clustering by enamel matrix proteins induces osteoblast differentiation through up-regulation of connexin 43 and N-cadherin. PLoS One.

[31] Zhang Y, Wei L, Miron RJ, Shi B, Bian Z (2015). Anabolic bone formation via a site-specific bone-targeting delivery system by interfering with semaphorin 4d expression. J Bone Miner Res Off J Am Soc Bone Miner Res.

[32] Zhang Y, Wu C, Friis T, Xiao Y (2010). The osteogenic properties of CaP/silk composite scaffolds. Biomaterials.

[33] Chan O, Coathup MJ, Nesbitt A, Ho CY, Hing KA, Buckland T, Campion C, Blunn GW (2012). The effects of microporosity on osteoinduction of calcium phosphate bone graft substitute biomaterials. Acta Biomater.

[34] Idowu B, Cama G, Deb S, Di Silvio L (2014). In vitro osteoinductive potential of porous monetite for bone tissue engineering. J Tissue Eng.

[35] Li J, Habibovic P, Yuan H, van den Doel M, Wilson CE, de Wijn JR, van Blitterswijk CA, de Groot K (2007). Biological performance in goats of a porous titanium alloy-biphasic calcium phosphate composite. Biomaterials.

[36] Barbieri D, Renard AJ, de Bruijn JD, Yuan H (2010). Heterotopic bone formation by nano-apatite containing poly(D, L-lactide) composites. Eur Cell Mater.

[37] Luo X, Barbieri D, Davison N, Yan Y, de Bruijn JD, Yuan H (2014). Zinc in calcium phosphate mediates bone induction: in vitro and in vivo model. Acta Biomater.

[38] Yuan H, Fernandes H, Habibovic P, de Boer J, Barradas AM, de Ruiter A, Walsh WR, van Blitterswijk CA, de Bruijn JD (2010). Osteoinductive ceramics as a synthetic alternative to autologous bone grafting. Proc Natl Acad Sci U S A.

[39] Dahlin C, Obrecht M, Dard M, Donos N. Bone tissue modelling and remodelling following guided bone regeneration in combination with biphasic calcium phosphate materials presenting different microporosity. Clin Oral Implants Res. 2015;26(7):814-22. 10.1111/clr.12361 24593049

[40] Yip I, Ma L, Mattheos N, Dard M, Lang NP. Defect healing with various bone substitutes. Clin Oral Implants Res. 2015;26(5):606-14. 10.1111/clr.12395 24702244

[41] Kimble KM, Eber RM, Soehren S, Shyr Y, Wang H-L (2004). Treatment of gingival recession using a collagen membrane with or without the use of demineralized freeze-dried bone allograft for space maintenance. J Periodontol.

[42] Miron RJ, Bosshardt DD (2016). OsteoMacs: Key players around bone biomaterials. Biomaterials.

